# OpenZika: An IBM World Community Grid Project to Accelerate Zika Virus Drug Discovery

**DOI:** 10.1371/journal.pntd.0005023

**Published:** 2016-10-20

**Authors:** Sean Ekins, Alexander L. Perryman, Carolina Horta Andrade

**Affiliations:** 1 Collaborations Pharmaceuticals, Inc., Fuquay-Varina, North Carolina, United States of America; 2 Department of Pharmacology, Physiology and Neuroscience, Rutgers University New Jersey Medical School, Newark, New Jersey, United States of America; 3 LabMol Laboratory for Molecular Modeling and Drug Design, Faculdade de Farmácia, Universidade Fedral de Goiás, Goiânia, Goiás, Brazil; George Washington University School of Medicine and Health Sciences, UNITED STATES

## Abstract

The Zika virus outbreak in the Americas has caused global concern. To help accelerate this fight against Zika, we launched the OpenZika project. OpenZika is an IBM World Community Grid Project that uses distributed computing on millions of computers and Android devices to run docking experiments, in order to dock tens of millions of drug-like compounds against crystal structures and homology models of Zika proteins (and other related flavivirus targets). This will enable the identification of new candidates that can then be tested in vitro, to advance the discovery and development of new antiviral drugs against the Zika virus. The docking data is being made openly accessible so that all members of the global research community can use it to further advance drug discovery studies against Zika and other related flaviviruses.

The Zika virus (ZIKV) has emerged as a major public health threat to the Americas as of 2015 [1]. We have previously suggested that it represents an opportunity for scientific collaboration and open scientific exchange [2]. The health of future generations may very well depend on the decisions we make, our willingness to share our findings quickly, and open collaboration to rapidly find a cure for this disease. Since February 1, 2016, when the World Health Organization deemed the cluster of microcephaly cases, Guillain-Barré, and other neurological disorders associated with ZIKV in Latin America and the Caribbean as constituting a Public Health Emergency of International Concern [3] (PHEIC), we have seen a rapid increase in publications (S1 References and main references). We [2] and others [4,5] described steps that could be taken to initiate a drug discovery program on ZIKV. For example, computational approaches, such as virtual screening of chemical libraries or focused screening to repurpose FDA and/or EU-approved drugs, can be used to help accelerate the discovery of an anti-ZIKV drug. An antiviral drug discovery program can be initiated using structure-based design, based on homology models of the key ZIKV proteins. With the lack of structural information regarding the proteins of ZIKV, we built homology models for all the ZIKV proteins, based on close homologs such as dengue virus, using freely available software [6] (S1 Table). These were made available online on March 3, 2016. We also predicted the site of glycosylation of glycoprotein E as Asn154, which was recently experimentally verified [7].

Since the end of March 2016, we have now seen two cryo-EM structures and 16 crystal structures of five target classes (S1 Table). These structures, alongside the homology models, represent potential starting points for docking-based virtual screening campaigns to help find molecules that are predicted to have high affinity with ZIKV proteins. These predictions can then be tested against the virus in cell-based assays and/or using individual protein-based assays. There are millions of molecules available that can be assayed, but which ones are likely to work, and how should we prioritize them?

In March, we initiated a new open collaborative project called OpenZika (Fig 1), with IBM’s World Community Grid (WCG, worldcommunitygrid.org), which has been used previously for distributed computing projects (S2 Table). On May 18, 2016, the OpenZika project began the virtual screening of ~6 million compounds that are in the ZINC database (Fig 1), as well as the FDA-approved drugs and the NIH clinical collection, using AutoDock Vina and the homology models and crystal structures (S1 Table, S1 Text, S1 References), to discover novel candidate compounds that can potentially be developed into new drugs for treating ZIKV. These will be followed by additional virtual screens with a new ZINC library of ~38 million compounds, and the PubChem database (at most ~90 million compounds), after their structures are prepared for docking.

**Fig 1:**
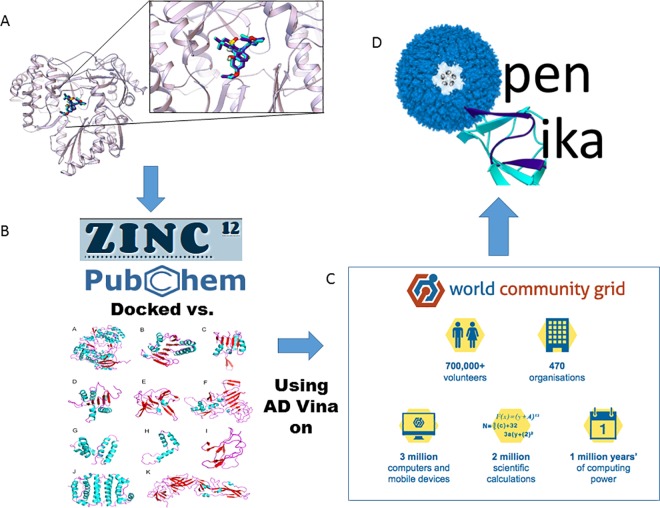
Workflow for the OpenZika project. A. Docking input files of the targets and ligands are prepared, and positive control docking studies are performed. The crystallographic binding mode of a known inhibitor is shown as sticks with dark purple carbon atoms, while the docked binding mode against the NS5 target from HCV has cyan carbons. Our pdbqt files of the libraries of compounds we screen are also openly accessible (http://zinc.docking.org/pdbqt/). B. We have already prepared the docking input files for ~6 million compounds from ZINC (i.e., the libraries that ALP previously used in the GO Fight Against Malaria project on World Community Grid), which are currently being used in the initial set of virtual screens on OpenZika. C. IBM’s World Community Grid is an internet-distributed network of millions of computers (Mac, Windows, and Linux) and Android-based tablets or smartphones in over 80 countries. Over 715,000 volunteers donate their dormant computer time (that would otherwise be wasted) towards different projects that are both (a) run by an academic or nonprofit research institute, and (b) are devoted to benefiting humanity. D. OpenZika is harnessing World Community Grid to dock millions of commercially available compounds against multiple ZIKV homology models and crystal structures (and targets from related viruses) using AutoDock Vina (AD Vina). This ultimately produces candidates (virtual hits that produced the best docking scores and displayed the best interactions with the target during visual inspection) against individual proteins, which can then be prioritized for in vitro testing by collaborators. After it is inspected, all computational data against ZIKV targets will be made open to the public on our website (http://openzika.ufg.br/experiments/#tab-id-7), and OpenZika results are also available upon request. The computational and experimental data produced will be published as quickly as possible.

Initially, compounds are being screened against the ZIKV homologs of drug targets that have been well-validated in research against dengue and hepatitis C viruses, such as NS5 and Glycoprotein E (S1 Table, S1 Text, S1 References). These may allow us to identify broad-spectrum antivirals against multiple flaviviruses, such as dengue virus, West Nile virus, and yellow fever virus. In addition, docking against the crystal structure of a related protein from a different pathogen can sometimes discover novel hits against the pathogen of interest [8].

As well as applying docking-based filters, the compounds virtually screened on OpenZika will also be filtered using machine learning models (S1 Text, S1 References). These should be useful selection criteria for subsequent tests by our collaborators in whole-cell ZIKV assays, to verify their antiviral activity for blocking ZIKV infection or replication. Since all OpenZika docking data will be in the public domain soon after they are completed and verified, we and other labs can then advance the development of some of these new virtual candidates into experimentally validated hits, leads, and drugs through collaborations with wet labs.

This exemplifies open science, which should help scientists around the world as they address the long and arduous process of discovering and developing new drugs. Screening millions of compounds against many different protein models in this way would take far more resources and time than any academic researcher could generally obtain or spend. As of August 16, 2016, we have submitted 894 million docking jobs. Over 6,934 CPU years have been donated to us, enabling over 439 million different docking jobs. We recently selected an initial batch of candidates for NS3 helicase (data openly available at http://openzika.ufg.br/experiments/#tab-id-7), for in vitro testing. Without the unique community of volunteers and tremendous resources provided by World Community Grid, this project would have been very difficult to initiate in a reasonable time frame at this scale.

The OpenZika project will ultimately generate several billion docking results, which could make it the largest computational drug discovery project ever performed in academia. The potential challenges we foresee will be finding laboratories with sufficient funding to pursue compounds, synthesize analogs, and develop target-based assays to validate our predictions and generate SAR (Structure-Activity Relationship) data to guide the process of developing the new hits into leads and then drugs. Due to the difficult nature of drug discovery and the eventual evolution of drug resistance, funding of ZIKV research once initiated will likely need to be sustained for several years, if not longer (e.g., HIV research has been funded for decades). As with other WCG projects, once scientists identify experimentally validated leads, finding a company to license them and pursue them in clinical trials and beyond will need incentives such as the FDA Tropical Disease Priority voucher, [9] which has a financial value on the open market [10].

By working together and opening our research to the scientific community, many other labs will also be able to take promising molecular candidates forward to accelerate progress towards defeating the ZIKV outbreak. We invite any interested researcher to join us (send us your models or volunteer to assay the candidates we identify through this effort against any of the flaviviruses), and we hope new volunteers in the general public will donate their dormant, spare computing cycles to this cause. We will ultimately report the full computational and experimental results of this collaboration.

**Advantages and Disadvantages of OpenZika**

Advantages

- Open Science could accelerate the discovery of new antivirals using docking and virtual screening
- Docking narrows down compounds to test, which saves time and money
- Free to use distributed computing on World Community Grid, and the workflow is simpler than using conventional supercomputers

Disadvantages

- Concern around intellectual property ownership and whether companies will develop drugs coming from effort
- Need for experimental assays will always be a factor
- Testing in vitro and in vivo is not free, nor are the samples of the compounds

## Supporting Information

S1 Text Information on OpenZika. (DOCX)

S1 Table Table of protein structures, PDB, and models to be used as docking targets. (DOCX)

S2 Table Select projects from the IBM World Community Grid (WCG) that use AutoDock Vina. (DOCX)

S1 References (DOCX)
