# Supplementary material for: OpenZika: An IBM World Community Grid Project to Accelerate Zika Virus Drug Discovery
Source: PLoS Negl Trop Dis. 2016 Oct 20;10(10):e0005023. doi: 10.1371/journal.pntd.0005023 (PMC5072634; doi:10.1371/journal.pntd.0005023)
Supplement: S1 Text — (DOCX) [file pntd.0005023.s001.docx]

**Supporting Information**

**Viewpoint**

**OpenZika: An IBM World Community Grid Project to Accelerate Zika Virus Drug Discovery**

Sean Ekins^1*^, Alexander L. Perryman^2*^ and Carolina Horta Andrade^3*^

## ^1^ Collaborations Pharmaceuticals, Inc., 5616 Hilltop Needmore Road, Fuquay-Varina, North Carolina 27526, United States.

^2^ Department of Pharmacology, Physiology and Neuroscience, Rutgers University–New Jersey Medical School, Newark, New Jersey 07103, United States.

**^3^** LabMol - Laboratory for Molecular Modeling and Drug Design, Faculdade de Farmácia, Universidade Federal de Goiás, Goiânia, Goiás 74605-170, Brazil.

**Email:** Carolina Horta Andrade: [andradech@yahoo.com](mailto:andradech@yahoo.com), Alexander L. Perryman: [Alex.L.Perryman@njms.rutgers.edu](mailto:Alex.L.Perryman@njms.rutgers.edu), Sean Ekins: [ekinssean@yahoo.com](mailto:ekinssean@yahoo.com)

**Running title:** OpenZika

S1 Text. Information on OpenZika

Since early 2016, the number of publications on Zika has increased dramatically, and these have strengthened the link of these disorders with ZIKV,[1-3] identified a potential entry receptor as AXL in neural stem cells [4] and demonstrated *in vitro* and *in vivo* models that could be used for testing potential small molecule therapeutics and vaccines.[5] At least one preliminary study has suggested that chloroquine may have some low micromolar activity *in vitro.*[6] A recent screen of a subset of FDA approved drugs identified low μM inhibitors, but 20/23 compounds would likely not be suitable for treating pregnant women; [7] therefore, there is a need to discover more potent compounds that are also safe during pregnancy.

More detail on the launch of OpenZika can be found from several press releases, [8-10] and data will be openly accessible to the scientific community (http://openzika.ufg.br/experiments/). Unlike previous WCG projects that offered to share data on request, this represents the first time that a project of this type is releasing the docking data to the community once it has been performed and inspected, by linking the Zika results to the project’s website.

Regarding the compounds being docked in OpenZika, more background information on the ZINC database can be found in two key publications.[11,12] The docking input (pdbqt) files we prepared for the initial libraries of ~6 million ligands (i.e., which we previously prepared for the GO Fight Against Malaria project) are all publicly available at <http://zinc.docking.org/pdbqt>. The NIH clinical collection is broadly accessible and contains drugs and clinical candidates.[13] The PubChem database has also been described elsewhere.[14,15] OpenZika uses AutoDock Vina software which has been described in great detail elsewhere.[16-18] We will filter molecules that are not FDA approved drugs using machine learning models developed for predicting some pharmacokinetic and toxicity properties, including metabolic stability [19-21] and hERG blockage [22,23] as examples, to try to increase the probability of finding compounds that can advance to the *in vivo* testing phase.
