# Supplementary material for: OpenZika: An IBM World Community Grid Project to Accelerate Zika Virus Drug Discovery
Source: PLoS Negl Trop Dis. 2016 Oct 20;10(10):e0005023. doi: 10.1371/journal.pntd.0005023 (PMC5072634; doi:10.1371/journal.pntd.0005023)
Supplement: S1 Table — (DOCX) [file pntd.0005023.s002.docx]

**Supporting Information**

**Viewpoint**

**OpenZika: An IBM World Community Grid Project to Accelerate Zika Virus Drug Discovery**

Sean Ekins^1*^, Alexander L. Perryman^2*^ and Carolina Horta Andrade^3*^

## ^1^ Collaborations Pharmaceuticals, Inc., 5616 Hilltop Needmore Road, Fuquay-Varina, North Carolina 27526, United States.

^2^ Department of Pharmacology, Physiology and Neuroscience, Rutgers University–New Jersey Medical School, Newark, New Jersey 07103, United States.

**^3^** LabMol - Laboratory for Molecular Modeling and Drug Design, Faculdade de Farmácia, Universidade Federal de Goiás, Goiânia, Goiás 74605-170, Brazil.

**Email:** Carolina Horta Andrade: [andradech@yahoo.com](mailto:andradech@yahoo.com), Alexander L. Perryman: [Alex.L.Perryman@njms.rutgers.edu](mailto:Alex.L.Perryman@njms.rutgers.edu), Sean Ekins: [ekinssean@yahoo.com](mailto:ekinssean@yahoo.com)

**Running title:** OpenZika

S1 Table. Table of protein structures, PDB and models to be used as docking targets.

| **Protein models or crystal structures ^a^** | **FASTA** | **Reference** |
| --- | --- | --- |
| NS5  (Virtual screens on OpenZika against this class have nearly completed; structures of homologs suggest location of two ligand binding sites) | [AHZ13508.1 (2,772..3,412)](https://www.ncbi.nlm.nih.gov/protein/AHZ13508.1?report=fasta&from=2772&to=3412) | [24] |
| FtsJ  (structures of homologs suggest location of ligand binding site) | [AHZ13508.1 (2,575..2,746)](https://www.ncbi.nlm.nih.gov/protein/AHZ13508.1?report=fasta&from=2575&to=2746) | [24] |
| NS4A ^b^ | [AHZ13508.1 (2,124..2,267)](https://www.ncbi.nlm.nih.gov/protein/AHZ13508.1?report=fasta&from=2124&to=2267) | [24] |
| HELICc | [AHZ13508.1 (1,859..1,975)](https://www.ncbi.nlm.nih.gov/protein/AHZ13508.1?report=fasta&from=1859&to=1975) | [24] |
| Peptidase S  (structures of homologs suggest location of ligand binding site) | [AHZ13508.1 (1,520..1,670)](https://www.ncbi.nlm.nih.gov/protein/AHZ13508.1?report=fasta&from=1520&to=1670) | [24] |
| DEXDc | [AHZ13508.1 (1,683..1,828)](https://www.ncbi.nlm.nih.gov/protein/AHZ13508.1?report=fasta&from=1683&to=1828) | [24] |
| NS2B ^b^ | [AHZ13508.1 (1,376..1,502)](https://www.ncbi.nlm.nih.gov/protein/AHZ13508.1?report=fasta&from=1376&to=1502) | [24] |
| NS2A ^b^ | [AHZ13508.1 (1,158..1,372)](https://www.ncbi.nlm.nih.gov/protein/AHZ13508.1?report=fasta&from=1158&to=1372) | [24] |
| NS1 | [AHZ13508.1 (796..1,148)](https://www.ncbi.nlm.nih.gov/protein/AHZ13508.1?report=fasta&from=796&to=1148) | [24] |
| E stem | [AHZ13508.1 (698..794)](https://www.ncbi.nlm.nih.gov/protein/AHZ13508.1?report=fasta&from=698&to=794) | [24] |
| Glycoprotein M | [AHZ13508.1 (216..290)](https://www.ncbi.nlm.nih.gov/protein/AHZ13508.1?report=fasta&from=216&to=290) | [24] |
| Propep | [AHZ13508.1 (126..214)](https://www.ncbi.nlm.nih.gov/protein/AHZ13508.1?report=fasta&from=126&to=214) | [24] |
| Capsid | [AHZ13508.1 (6..122)](https://www.ncbi.nlm.nih.gov/protein/AHZ13508.1?report=fasta&from=6&to=122) | [24] |
| Glycoprotein E | [AHZ13508.1 (291..592)](https://www.ncbi.nlm.nih.gov/protein/AHZ13508.1?report=fasta&from=291&to=592)  [AHZ13508.1 (601..693)](https://www.ncbi.nlm.nih.gov/protein/AHZ13508.1?report=fasta&from=601&to=693) | [24,25] |
| NS4B ^b^ | [AHZ13508.1 (2,270..2,514)](https://www.ncbi.nlm.nih.gov/protein/AHZ13508.1?report=fasta&from=2270&to=2514) | [24] |
| Glycoprotein E ^c^  **PDB 5IRE, 5IZ7, 5JHL** (Antibody-bound) and **5JHM** |  | [26-28] |
| Helicase domain of NS3  **PDB 5JMT** (5JPS = not yet released)  Structures of homologs suggest location of two ligand binding sites; virtual screens against this target class are currently ongoing on OpenZika |  | [29] |
| NS1  (C-terminal fragment; host-interaction protein)  **PDB 5IY3**  Structures of homologs suggest location of ligand binding site; virtual screens against this class are currently being prepared |  | [28] |

a) If the entry does not have a note regarding “structures of homologs suggest location of ligand binding site,” then this information should be considered as “to be determined” (after further analyses of the structures of the homologs have been performed). We are also docking compounds against the crystal structures of the virus templates that were used to create these homology models and additional crystal structures of proteins (from pathogens) that have high sequence identity to the ZIKV targets.

b) Because of low GMQE scores and/or low coverage in sequence identity with the template, these homology models of ZIKV proteins are considered to have lower accuracy and/or could not be validated

c) Both the mature and immature protein conformers of Glycoprotein E can be used as targets, to study how this affects the nature of the different hits identified.
