# Supplementary material for: OpenZika: An IBM World Community Grid Project to Accelerate Zika Virus Drug Discovery
Source: PLoS Negl Trop Dis. 2016 Oct 20;10(10):e0005023. doi: 10.1371/journal.pntd.0005023 (PMC5072634; doi:10.1371/journal.pntd.0005023)
Supplement: S2 Table — (DOCX) [file pntd.0005023.s003.docx]

**Supporting Information**

**Viewpoint**

**OpenZika: An IBM World Community Grid Project to Accelerate Zika Virus Drug Discovery**

Sean Ekins^1*^, Alexander L. Perryman^2*^ and Carolina Horta Andrade^3*^

## ^1^ Collaborations Pharmaceuticals, Inc., 5616 Hilltop Needmore Road, Fuquay-Varina, North Carolina 27526, United States.

^2^ Department of Pharmacology, Physiology and Neuroscience, Rutgers University–New Jersey Medical School, Newark, New Jersey 07103, United States.

**^3^** LabMol - Laboratory for Molecular Modeling and Drug Design, Faculdade de Farmácia, Universidade Federal de Goiás, Goiânia, Goiás 74605-170, Brazil.

**Email:** Carolina Horta Andrade: [andradech@yahoo.com](mailto:andradech@yahoo.com), Alexander L. Perryman: [Alex.L.Perryman@njms.rutgers.edu](mailto:Alex.L.Perryman@njms.rutgers.edu), Sean Ekins: [ekinssean@yahoo.com](mailto:ekinssean@yahoo.com)

**Running title:** OpenZika

S2 Table. Select projects from the IBM World Community Grid (WCG) that use AutoDock Vina. WCG is enabled by Berkeley Open Infrastructure for Network Computing (BOINC) [30], and with support from the [National Science Foundation](http://www.nsf.gov/) [31]. WCG has helped researchers identify potential drugs or new chemical tools for the following diseases.

| **Disease/ Project Name** | **Details** | **Reference** |
| --- | --- | --- |
| FightAIDS@Home and FightAIDS@Home2 | The first phase of the project made significant advances in HIV research. As the virus evolves, the research team is now pioneering the use of new analysis techniques (BEDAM-based re-scoring of the compounds docked in phase 1)[32-34]to better identify promising anti-HIV drugs in phase 2. | http://fightaidsathome.scripps.edu/ |
| Malaria (GO Fight Against Malaria) | The project aimed to discover promising candidates that could be developed into new drugs that cure drug-resistant forms of malaria (with some experiments that also targeted extensively drug-resistant tuberculosis). A lack of grant funding is slowing down the analysis and experimental assays of the GO FAM results, but some novel inhibitors have already been discovered. | [35] [17]  <http://GOFightAgainstMalaria.scripps.edu> |
| Leishmaniasis (Drug Search for Leishmaniasis) | The aim of the project is to identify potential drug candidates that could possibly be developed into treatments for Leishmaniasis. | [36] |
| Schistosomiasis (Say No to Schistosoma) | The mission of the project is to identify potential drug candidates that could possibly be developed into treatments for schistosomiasis. | [37] |
| Ebola (Outsmart Ebola Together) | This project’s goal is to develop an antiviral active against Ebola | [38] |
