# Supplementary material for: OpenZika: An IBM World Community Grid Project to Accelerate Zika Virus Drug Discovery
Source: PLoS Negl Trop Dis. 2016 Oct 20;10(10):e0005023. doi: 10.1371/journal.pntd.0005023 (PMC5072634; doi:10.1371/journal.pntd.0005023)
Supplement: S1 References — (DOCX) [file pntd.0005023.s004.docx]

**Supporting Information**

**Viewpoint**

**OpenZika: An IBM World Community Grid Project to Accelerate Zika Virus Drug Discovery**

Sean Ekins^1*^, Alexander L. Perryman^2*^ and Carolina Horta Andrade^3*^

## ^1^ Collaborations Pharmaceuticals, Inc., 5616 Hilltop Needmore Road, Fuquay-Varina, North Carolina 27526, United States.

^2^ Department of Pharmacology, Physiology and Neuroscience, Rutgers University–New Jersey Medical School, Newark, New Jersey 07103, United States.

**^3^** LabMol - Laboratory for Molecular Modeling and Drug Design, Faculdade de Farmácia, Universidade Federal de Goiás, Goiânia, Goiás 74605-170, Brazil.

**Email:** Carolina Horta Andrade: [andradech@yahoo.com](mailto:andradech@yahoo.com), Alexander L. Perryman: [Alex.L.Perryman@njms.rutgers.edu](mailto:Alex.L.Perryman@njms.rutgers.edu), Sean Ekins: [ekinssean@yahoo.com](mailto:ekinssean@yahoo.com)

**Running title:** OpenZika

S1 References

1. Rasmussen SA, Jamieson DJ, Honein MA, Petersen LR. Zika Virus and Birth Defects--Reviewing the Evidence for Causality. N Engl J Med. 2016; 374:1981-7.

2. Mlakar J, Korva M, Tul N, Popovic M, Poljsak-Prijatelj M, Mraz J, et al. Zika Virus Associated with Microcephaly. N Engl J Med. 2016; 374:951-8.

3. Cugola FR, Fernandes IR, Russo FB, Freitas BC, Dias JLM, Guimaraes KP, et al. The Brazilian zika virus strain causes birth defects in experimental models. Nature. 2016; In Press.

4. Nowakowski TJ, Pollen AA, Di Lullo E, Sandoval-Espinosa C, Bershteyn M, Kriegstein AR. Expression Analysis Highlights AXL as a Candidate Zika Virus Entry Receptor in Neural Stem Cells. Cell Stem Cell. 2016; 18:591-6.

5. Shan C, Xie X, Muruato AE, Rossi SL, Roundy CM, Azar SR, et al. An Infectious cDNA Clone of Zika Virus to Study Viral Virulence, Mosquito Transmission, and Antiviral Inhibitors. Cell Host Microbe. 2016.

6. Delvecchio R, Higa LM, Pezzuto P, Valadao AL, Garcez PP, Monteiro FL, et al. Chloroquine inhibits Zika Virus infection in different cellular models. bioRxiv. 2016.

7. Barrows NJ, Campos RK, Powell ST, Prasanth KR, Schott-Lerner G, Soto-Acosta R, et al. A Screen of FDA-Approved Drugs for Inhibitors of Zika Virus Infection. Cell Host Microbe. 2016.

8. Anon. Zika Treatment Search Launched, Fueled By IBM's World Community Grid. 2016. <http://www-03.ibm.com/press/us/en/pressrelease/49778.wss>.

9. Andrade CH. Help an International Research Team Fight the Zika Virus. 2016. <https://secure.worldcommunitygrid.org/about_us/viewNewsArticle.do?articleId=480>.

10. Andrade CH, Perryman AL, Ekins S. OpenZika. 2016. <https://www.worldcommunitygrid.org/research/zika/overview.do>.

11. Irwin JJ, Shoichet BK. ZINC--a free database of commercially available compounds for virtual screening. J Chem Inf Model. 2005; 45:177-82.

12. Irwin JJ, Sterling T, Mysinger MM, Bolstad ES, Coleman RG. ZINC: a free tool to discover chemistry for biology. J Chem Inf Model. 2012; 52:1757-68.

13. Anon. NIH Clinical Collection. 2014. <http://www.nihclinicalcollection.com/>.

14. Kim S, Thiessen PA, Bolton EE, Chen J, Fu G, Gindulyte A, et al. PubChem Substance and Compound databases. Nucleic Acids Res. 2016; 44:D1202-13.

15. Wang Y, Bolton E, Dracheva S, Karapetyan K, Shoemaker BA, Suzek TO, et al. An overview of the PubChem BioAssay resource. Nucleic Acids Res. 2010; 38:D255-66.

16. Trott O, Olson AJ. AutoDock Vina: improving the speed and accuracy of docking with a new scoring function, efficient optimization, and multithreading. J Comput Chem. 2010; 31:455-61.

17. Perryman AL, Yu W, Wang X, Ekins S, Forli S, Li SG, et al. A Virtual Screen Discovers Novel, Fragment-Sized Inhibitors of Mycobacterium tuberculosis InhA. J Chem Inf Model. 2015.

18. Perryman AL, Santiago DN, Forli S, Santos-Martins D, Olson AJ. Virtual screening with AutoDock Vina and the common pharmacophore engine of a low diversity library of fragments and hits against the three allosteric sites of HIV integrase: participation in the SAMPL4 protein-ligand binding challenge. J Comput Aided Mol Des. 2014; 28:429-41.

19. Perryman AL, Stratton TP, Ekins S, Freundlich JS. Predicting Mouse Liver Microsomal Stability with "Pruned" Machine Learning Models and Public Data. Pharm Res. 2016; 33:433-49.

20. Clark AM, Dole K, Coulon-Spektor A, McNutt A, Grass G, Freundlich JS, et al. Open Source Bayesian Models. 1. Application to ADME/Tox and Drug Discovery Datasets. J Chem Inf Model. 2015; 55:1231-45.

21. Ekins S. Progress in computational toxicology. J Pharmacol Toxicol Methods. 2014; 69:115-40.

22. Braga RC, Alves VM, Silva MF, Muratov E, Fourches D, Tropsha A, et al. Tuning HERG out: antitarget QSAR models for drug development. Curr Top Med Chem. 2014; 14:1399-415.

23. Braga RC, Alves VM, Soilva MFB, Muratov E, Fourches D, Liao LM, et al. Pred-hERG: A Novel web-Accessible Computational Tool for Predicting Cardiac Toxicity. Mol Informatics. 2015; 34:698-701.

24. Ekins S, Liebler J, Neves BJ, Lewis WG, Coffee M, Bienstock R, et al. Illustrating and homology modeling the proteins of the Zika virus. F1000Res. 2016; 5:275.

25. Ekins S, Mietchen D, Coffee M, Stratton TP, Freundlich JS, Freitas-Junior L, et al. Open drug discovery for the Zika virus. F1000Res. 2016; 5:150.

26. Sirohi D, Chen Z, Sun L, Klose T, Pierson TC, Rossmann MG, et al. The 3.8 A resolution cryo-EM structure of Zika virus. Science. 2016; 352:467-70.

27. Kostyuchenko VA, Lim EX, Zhang S, Fibriansah G, Ng TS, Ooi JS, et al. Structure of the thermally stable Zika virus. Nature. 2016; 533:425-8.

28. Song H, Qi J, Haywood J, Shi Y, Gao GF. Zika virus NS1 structure reveals diversity of electrostatic surfaces among flaviviruses. Nat Struct Mol Biol. 2016; 23:456-8.

29. Tian H, Ji X, Yang X, Xie W, Yang K, Chen C, et al. The crystal structure of Zika virus helicase: basis for antiviral drug design. Protein Cell. 2016.

30. Anon. Open-source software for volunteer computing 2016. <http://boinc.berkeley.edu/>.

31. Anon. National Science Foundation. 2016. <http://www.nsf.gov/>.

32. Gallicchio E, Lapelosa M, Levy RM. The Binding Energy Distribution Analysis Method (BEDAM) for the Estimation of Protein-Ligand Binding Affinities. J Chem Theory Comput. 2010; 6:2961-77.

33. Deng N, Forli S, He P, Perryman A, Wickstrom L, Vijayan RS, et al. Distinguishing binders from false positives by free energy calculations: fragment screening against the flap site of HIV protease. J Phys Chem B. 2015; 119:976-88.

34. Gallicchio E, Deng N, He P, Wickstrom L, Perryman AL, Santiago DN, et al. Virtual screening of integrase inhibitors by large scale binding free energy calculations: the SAMPL4 challenge. J Comput Aided Mol Des. 2014; 28:475-90.

35. Anon. GO fight against malaria. 2016. <http://www.worldcommunitygrid.org/research/gfam/overview.do>.

36. Anon. Drug search for leishmaniasis. 2016. <http://www.worldcommunitygrid.org/research/dsfl/overview.do>.

37. Anon. Say no to schistosoma. 2016. <http://www.worldcommunitygrid.org/research/sn2s/overview.do>.

38. Anon. Outsmart Ebola together. 2016. <http://www.worldcommunitygrid.org/research/oet1/overview.do>.
